# A scalable and transparent data pipeline for AI-enabled health data ecosystems

**DOI:** 10.3389/fmed.2024.1393123

**Published:** 2024-07-30

**Authors:** Tuncay Namli, Ali Anıl Sınacı, Suat Gönül, Cristina Ruiz Herguido, Patricia Garcia-Canadilla, Adriana Modrego Muñoz, Arnau Valls Esteve, Gökçe Banu Laleci Ertürkmen

**Affiliations:** ^1^SRDC Software Research Development and Consultancy A. Ş., Ankara, Turkey; ^2^Fundacio Sant Joan De Deu, Barcelona, Spain

**Keywords:** artificial intelligence, dataset, harmonization, transparency, FHIR, interoperability, health data spaces

## Abstract

**Introduction:**

Transparency and traceability are essential for establishing trustworthy artificial intelligence (AI). The lack of transparency in the data preparation process is a significant obstacle in developing reliable AI systems which can lead to issues related to reproducibility, debugging AI models, bias and fairness, and compliance and regulation. We introduce a formal data preparation pipeline specification to improve upon the manual and error-prone data extraction processes used in AI and data analytics applications, with a focus on traceability.

**Methods:**

We propose a declarative language to define the extraction of AI-ready datasets from health data adhering to a common data model, particularly those conforming to HL7 Fast Healthcare Interoperability Resources (FHIR). We utilize the FHIR profiling to develop a common data model tailored to an AI use case to enable the explicit declaration of the needed information such as phenotype and AI feature definitions. In our pipeline model, we convert complex, high-dimensional electronic health records data represented with irregular time series sampling to a flat structure by defining a target population, feature groups and final datasets. Our design considers the requirements of various AI use cases from different projects which lead to implementation of many feature types exhibiting intricate temporal relations.

**Results:**

We implement a scalable and high-performant feature repository to execute the data preparation pipeline definitions. This software not only ensures reliable, fault-tolerant distributed processing to produce AI-ready datasets and their metadata including many statistics alongside, but also serve as a pluggable component of a decision support application based on a trained AI model during online prediction to automatically prepare feature values of individual entities. We deployed and tested the proposed methodology and the implementation in three different research projects. We present the developed FHIR profiles as a common data model, feature group definitions and feature definitions within a data preparation pipeline while training an AI model for “predicting complications after cardiac surgeries”.

**Discussion:**

Through the implementation across various pilot use cases, it has been demonstrated that our framework possesses the necessary breadth and flexibility to define a diverse array of features, each tailored to specific temporal and contextual criteria.

## 1 Introduction

### 1.1 Background and objectives

Transparency, and traceability are considered among the key requirements for trustworthy artificial intelligence (AI) by the AI-Act which will be governing the use of AI solutions in the EU (1). Lack of transparency in the data preparation process, i.e., the difficulty in tracking and understanding the transformations and manipulations that the data undergoes before being used for training is a major issue for building trustworthy AI solutions (2).

Today’s AI models depend on a complex, iterative process involving extensive communication among medical professionals, data scientists, and database administrators. Medical experts outline the specific data needed for the AI project to data scientists and AI developers, who then pass these requirements to database administrators. These administrators are responsible for retrieving the relevant data from existing sources, such as Electronic Health Records (EHR), based on the defined variables. Typically, this procedure is manually carried out by database administrators, resulting in time-consuming, labor-intensive tasks that lack transparency and traceability. Data scientists check the accuracy and relevance of the data, while medical professionals evaluate the performance of the AI model trained using this data. This prone-to-error and laborious back-and-forth continues until there is a mutual understanding and satisfaction with the data prepared for the AI application. This lack of transparency can lead to several issues:

• Reproducibility: Without knowing the exact steps taken during data preparation, it becomes difficult to reproduce the same dataset or validate the results obtained from the AI model. This also hampers the ability to effectively train AI models across several sites via a federated learning architecture. For example, researchers may decide to exclude data from certain patients with specific phenotypes (e.g., having a condition like epilepsy). Even if they document this exclusion by indicating the name of the disease, the lack of clear coding for exclusion criteria can still pose a problem due to insufficient transparency. Medical terminologies (e.g., ICD-10 codes for diagnosis) are used to indicate phenotypes, and the usage of these medical concepts can vary among different healthcare settings. Even if the same terminology is used, the practical definition of the phenotype can differ between two healthcare settings. Therefore, when an AI model is deployed in a different setting, it is crucial that the phenotype definitions are clear and transparent. This allows for proper configuration and customization of data mapping or preprocessing steps according to how medical terminologies are used in that specific setting.
• Debugging: When unexpected results occur during model training or inference, it can be challenging to identify the root cause without knowing how the training data was prepared.
• Bias and Fairness: Data transformations and preprocessing steps can inadvertently introduce biases into the dataset, leading to biased AI models. During training data extraction and data cleaning step, decisions on how to handle missing values can introduce biases. For example, if data for certain racial groups is more likely to have missing values, imputing these with overall mean values might not accurately reflect the health status of these groups. Then suppose that an AI model trained and deployed to predict health risks for a diverse patient population where this data cleaning step not documented transparently. Because the model was trained on a biased dataset, it may not perform well for these underrepresented groups. Due to this unidentified bias, the model may perform well on the majority population but poorly on minorities which may exacerbate existing health disparities. Without traceability, it’s challenging to detect and mitigate these biases.
• Compliance and Regulation: In regulated domains such as healthcare, there are regulatory requirements for documenting data processing steps for transparency and auditability purposes.

In this paper, we propose a formal data preparation pipeline specification to overcome the limitations of the manual and error-prone data extraction processes for AI and data analytics applications, while also addressing the issue of traceability. We introduce a declarative JSON-based language designed for specifying how to extract data from datasets that adhere to Common Data Models, specifically those compatible with HL7 Fast Healthcare Interoperability Resources (FHIR) standards (3), as part of a pipeline process to prepare data for AI. Our goal is to enhance the scalability, transparency, and reproducibility of AI applications by streamlining the data extraction phase and clearly separating medical knowledge from data engineering knowledge. Our approach also endeavors to offer a practical methodology for realizing the objectives outlined in the European Health Data Space (EHDS) legislation (4) which aims to facilitate health data portability and foster the development of a unified market for health data for secondary use purposes. The transparent and declarative model used to define the data preparation pipeline supports the aggregation of health data from diverse sources, thereby making them accessible for clinical research.

Our objective is to establish a data preparation pipeline originating from Electronic Health Record (EHR) sources to generate AI-ready training datasets. This task presents several challenges due to the inherent complexity of EHRs, rendering them unsuitable for direct use as feature vectors in training AI models (5–7). EHR data is structured in intricate, nested, high-dimensional models with diverse data types often linked to external domain-specific terminologies and code systems, enhancing the semantic understanding of data entities. However, this structure doesn’t align directly with the flat feature vectors expected by AI methods, typically represented as normalized, domain-agnostic value sets. Moreover, EHR data records unevenly distributed clinical events, resulting in irregularly sampled sparse time series data, further complicated by the presence of missing values.

Converting EHR data into feature vectors suitable for AI methods necessitates multiple steps, involving various decisions. These decisions encompass selecting domain-specific codes from international code systems to determine which data entities from EHR to include, identifying necessary temporal joins and aggregations, determining the resampling strategy for longitudinal EHR data, specifying transformations for normalization, unit conversion, and harmonization, and devising approaches to handle missing data. The design of our declarative data preparation pipeline definition has been guided by these challenges. It is crafted to transparently define each step of the transformation pipeline as a sequence of data processing and transformation actions in a standardized manner.

Our data preparation pipeline model is technology agnostic; it provides a machine processable definition of the pipeline steps. In this paper, to demonstrate the effectiveness of the proposed methodology, we also briefly describe our implementation of an engine, called “onfhir-feast”, that processes this machine processable pipeline definition to extract AI-ready datasets from EHR sources. Implemented as a high-performance distributed engine, we showcase its ability to efficiently extract datasets for various use cases. This domain-specific, technology-agnostic language establishes a standardized approach for a variety of stakeholders, including data scientists, health data owners, and AI or clinical decision support service vendors, to collaborate and develop AI-based solutions or conduct research studies. This framework enables scalability and reproducibility, ensuring that solutions and studies can be effectively implemented and replicated across different healthcare settings.

### 1.2 Related research

One of the pioneering initiatives to enable observational research on top of EHRs is OHDSI (8). OHDSI offers the OMOP Common Data Model (CDM) (9), which standardizes the structure and content of observational data with the support of a standardized vocabulary. Additionally, OHDSI provides a suite of open-source tools, including ATLAS for designing and executing observational research studies, and ACHILLES for characterizing and visualizing source data. While OMOP CDM serves as a solid foundation, it requires extension and specialization to cater to the needs of domain specific research studies, such as cancer research (10) and medical imaging (11). In OMOP CDM approach, it is not possible to document these extensions and customizations in a machine processable and traceable manner. Our approach addresses this gap by introducing a FHIR-based CDM, which meticulously documents all customizations in a machine-processable manner via the profiling methodology. Although OHDSI’s open-source tools facilitate population queries and dataset extraction, this process is not documented in a machine processable manner which diminishes the end-to-end transparency, and traceability of the dataset preparation process. This deficiency hampers reproducibility and auditability, key requirements of AI-Act.

There have been a number of efforts in the literature to flatten the hierarchical EHR data to create AI-ready tabular datasets. Fiddle (6) provides an open-source generic preprocessing pipeline implementation for extracting structured data from the EHR data with three distinct steps, namely for pre-filtering, transforming and post-filtering. As HL7 FHIR is widely supported by numerous health care institutions and vendors of clinical information systems, several efforts focused on flattening EHR data represented as FHIR resources for extracting AI friendly data sets. Liu et al. (12) utilized the FHIR Bulk Data API to create population-level exports from clinical systems, into a file format often referred to as “Flat-FHIR’ represented in NDJSON-based data format. FHIR-DHP (5) proposes a generic data harmonization pipeline (DHP) that is composed of data exchange, mapping, and export operations to transform EHR data to FHIR standard first, then to a relational database format, and exporting the data to a custom flattened JSON format as an AI-friendly format. FhirExtinguisher (13) has extended the FHIR Search API with an additional projection layer using FHIRPath, to build a tool for transforming FHIR resources into tabular data. Pathling (14) proposes an extended FHIR Analytics API, as a specialization of the FHIR API that focuses on providing functionality useful for health data analytics applications, namely: importing bulk FHIR data, execution of aggregation-based queries across a data set, searching via FHIRPath queries for cohort selection and extracting datasets to create custom data extracts for input into other tools and workflow. Although these efforts provided a generic methodology (5, 12) and/or extended API specification and implementation (13, 14) to flatten EHR data as tabular data sets, they do not provide a declarative model to formally define the data preparation pipeline. Our technology agnostic approach complements these, by providing an additional level of abstraction for enabling transparency, traceability, and reproducibility of AI methods. It should be noted that these efforts such as (13, 14) can be utilized to implement a transformation engine implementing the declarative data preparation pipeline definition proposed in this paper.

## 2 Materials and methods

### 2.1 Common data modeling for AI use case

A pivotal component of our methodology involves the construction of Common Data Models (CDMs) tailored for AI applications. In our methodology, the CDMs are meticulously built utilizing the HL7 Fast Healthcare Interoperability Resources (FHIR) standard, leveraging the FHIR profiling technique. FHIR profiling is the process of defining or constraining FHIR resources to address specific requirements. This involves customizing FHIR’s generic, standardized resources to create more precise models that cater to particular use cases, workflows, or data exchange scenarios within healthcare applications. These profiles dictate how FHIR resources are used, including the elements they must contain, the cardinality of these elements (e.g., optional, mandatory, repeating), and value sets or data types for each element.

In healthcare, AI applications are generally built for specific use cases, and the data requirements, so called variables needed for executing the AI model or visualizing the results, are declared within those use cases. One of the primary benefits of utilizing a CDM with HL7 FHIR profiling is the creation of a customized standard data model that is specifically tailored to the unique requirements of each AI use case. By defining a machine processable CDM that precisely aligns with the specific data elements, structures, and terminologies relevant to the use case, we ensure that the AI system is built upon a solid foundation of accurate and relevant data.

Utilizing a CDM defined by the HL7 FHIR standard is the establishment of a standardized interface for querying and accessing health records. This standardization not only simplifies the integration of disparate health information systems but also ensures that AI algorithms can access the necessary data in a consistent and reliable manner. By facilitating a uniform method to search and retrieve health records, we significantly reduce the complexity and variability often encountered in health data, thus enabling more efficient data processing and analysis.

The use of HL7 FHIR in defining our CDM enables the explicit declaration of information critical to the AI use case, such as phenotype and AI feature definitions. This is achieved by referring to inherent FHIR structures and standardized medical terminologies through the value set references. With this approach, an AI use case transparently declares its information of interest. As a result, our methodology not only enhances the semantic interoperability of health data but also ensures that the AI systems have access to a rich and semantically coherent dataset. This level of specificity and clarity in data representation is essential for the development of AI algorithms that are both effective and reliable in clinical settings.

As a common data model for a specific analytic or AI use case, we propose to provide a FHIR Implementation Guide including the following machine processable FHIR based definitions.

• A FHIR CapabilityStatement defining the list of related FHIR resource types needed for this use case as well as references to search parameters to be used to search related data for each resource type.
• A list of StructureDefinition resources defining syntactic and semantic customizations and restrictions representing a category of health events or facts needed for the use case.
• A list of ValueSet and/or CodeSystem resources defining the relevant concepts from terminology systems for restricting certain elements value sets or define information of interest.
• The adoption of a Common Data Model based on the HL7 FHIR standard, tailored through the FHIR profiling approach, offers significant advantages for the development of AI in healthcare. It provides a standardized, customizable, and semantically rich framework for accessing and processing health records, thereby laying the groundwork for scalable and transparent AI solutions in healthcare.

### 2.2 Declarative model to define the data preparation pipeline

We propose an end-to-end data preparation pipeline that begins with clinical data sources, such as Electronic Health Records (EHRs) and supplies AI systems with training datasets. This pipeline can also be utilized to run intelligent clinical applications and decision support services built based on AI models readily on EHRs by seamlessly retrieving the input parameters.

By utilizing the Common Data Model built upon HL7 FHIR, we establish a standardized interface for accessing source data effortlessly. Our goal is to create a transparent pipeline utilizing this FHIR interface to generate a dataset optimized for AI applications. However, this presents a challenge: converting the nested, hierarchical data model of FHIR into a tabular or time series format compatible with mainstream AI frameworks such as TensorFlow (15), Pythorch (16) or Scikit-learn (17).

EHR data is intricate, featuring high dimensionality, irregular time series sampling, and a variety of data types with diverse representations of clinical events. Converting this complex EHR data into flat feature vectors that align with Machine Learning (ML) techniques poses several challenges.

• First and foremost, performing temporal joins and aggregations over EHR data is necessary to derive features or outcome variables in a dataset. These derived features will become columns in the tabular format expected by AI frameworks. This process also requires tailoring to the specific requirements of each use case. For instance, consider a scenario involving EHR data where a particular lab result, such as creatinine, is represented as a FHIR Observation type resource according to the FHIR standard. In a specific use case, like predicting complications after cardiac surgeries, various creatinine results may be relevant. These could include the creatinine level before surgery, the first creatinine result within 24 h after surgery, the latest creatinine result, the average of all results, and the difference between the first and last creatinine results. Each of these aspects needs to be defined as separate features specific to the given use case scenario. Thus, it’s essential to establish a method for defining how these use case-specific tabular feature sets can be extracted from the hierarchical, relational FHIR-based model for each unique use case scenario.
• Frequently, transformations are required to adjust the scale or discretize the numeric values found in EHR data, such as laboratory values. This is necessary to create normalized features that align with the expectations of ML models. Additionally, numeric values expressed in various units may need to be converted to a specified unit for the sake of harmonization.
• In EHR data, clinical events are logged as they occur within the clinical workflow, leading to irregular sampling of time series events, which differs from the regular sampling expected by ML methods. Consequently, it’s essential to establish strategies for resampling longitudinal EHR data to meet the requirements of specific use cases.

Each of these steps requires numerous decisions from data scientists in the data preparation process. Transparency within these decisions is vital for data transparency, as they heavily impact the characteristics, and quality of the resulting dataset. To ensure clarity in defining these steps or decisions, we introduce a declarative model aimed at precisely defining each step of the transformation in the pipeline from EHR data to AI-ready feature sets.

The HL7 FHIR API offers a standardized query language, included within the FHIR API’s search interaction, enabling the querying of health data. Additionally, there’s another language known as FHIRPath (18), designed for processing and navigating FHIR content. Our declarative model leverages these FHIR Query and FHIRPath statements to transparently define health datasets as a series of data processing and transformation steps in a standardized manner. Through this declarative model, transformation steps can be precisely defined and executed to prepare training, validation, or test datasets for AI. Moreover, it can also facilitate the preparation of features for executing decision support models during online prediction.

In the process of designing our declarative model, we aimed to recognize the steps typically taken by data scientists or research groups when creating a dataset through conventional methods, which often involve coding in Python and/or SQL. We endeavored to devise a practical approach to achieve the same using FHIR constructs. The following steps have been identified and form the primary sections of our declarative model:

• Definition of target population: This step entails identifying the target cohort by declaratively specifying the characteristics of the data entities that will comprise the target population for a specific use case, utilizing inclusion and exclusion criteria. In certain scenarios, entities eligible for inclusion in the dataset may be limited to specific time periods. This step also allows for defining these eligible time periods tailored to the specific use case requirements.
• Definition of feature groups: In this step, we define an intermediate result set, as a group of base features, that can be retrieved from EHR and can be utilized in the next step to calculate the final set of features required by the use case. At this step it is also possible to define transformations such as unit conversions to create harmonized data sets.
• Definition of final datasets: This step includes defining the individual features based on the base features identified in feature groups. At this step, we first define the rules for resampling of longitudinal health data, and also define anchor time points that are important for the use case. Following this, we declaratively specify how final features in the dataset can be calculated based on the base features, and anchor timepoints, through a set of temporal and contextual constraints, aggregations, and transformations.

In the subsequent sections, we explore the intricacies of the methodologies and processes involved, elucidating the benefits and functionalities of the suggested approach and solution. Through the application of the proposed language, we offer exemplary definitions to demonstrate the adaptability of our method across a diverse range of use cases.

#### 2.2.1 Definition of target population

The initial phase in preparing the dataset involves defining the target cohort, which entails specifying the characteristics or phenotype of the entities (for instance, patients) designated as the target population for the current use case, and whose information will be incorporated into the dataset. In this context, we adhere to the definition provided by OHDSI, which describes a cohort as “a set of persons who satisfy one or more inclusion criteria for a duration of time” (19).

In our approach, a single population definition is engineered for versatility across various use cases, thereby allowing it to be a distinct, reusable component within different dataset definitions. For illustration, Table 1 presents a population definition tailored for datasets focusing on Parkinson’s disease patients. Each construct, starting with the population definition, is initiated with metadata elements such as title, description, version, and a canonical URL, to provide a comprehensive overview of the definition. To establish a population definition effectively, it is essential first to identify the specific entities comprising the population. Within the FHIR framework, there are distinct resource types—such as Patient, Practitioner, and Organization—designed to represent individuals (e.g., patients or healthcare practitioners) or entities (e.g., organizations), along with their foundational information. All ancillary resources link back to these primary resources to delineate their interrelations. For instance, a lab result, denoted by a FHIR Observation resource, specifies its associated patient through a reference type element that points to the pertinent FHIR Patient resource.

**TABLE 1:** An example definition of target population for a simple use case; “Patients diagnosed with Parkinson.”

{
”url”: “https://aiccelerate.eu/cohorts/pilot2/parkinson_cohort”,
”name”: “parkinson_cohort”,
”title”: “Patients diagnosed with Parkinson”,
”description”: “Patients diagnosed with Parkinson (ICD-10 G20 code)”,
”version”: “0.1”,
”date”: “2022-04-21T00:00:00”,
”fhirVersion”: “4.0.1”,
”publisher”: “AICCELERATE WP1 Team (SRDC Corp.)”,
”entityType”: [”Patient”],
”eligibilityCriteria”: [
{
“fhirSearch”: “?”,
“description”: “All patients with a parkinson diagnosis (ICD-10 G20)”,
“filter”: [
{
“resourceType”: “Condition”,
“fhirSearch”: “?code = http://hl7.org/fhir/sid/icd-10| G20&patient = {{Patient}}”,
“entities”: [”Condition.subject”],
“eventTime”: “Condition.onsetDateTime”
}
]
}
]

**FIGURE 1:**
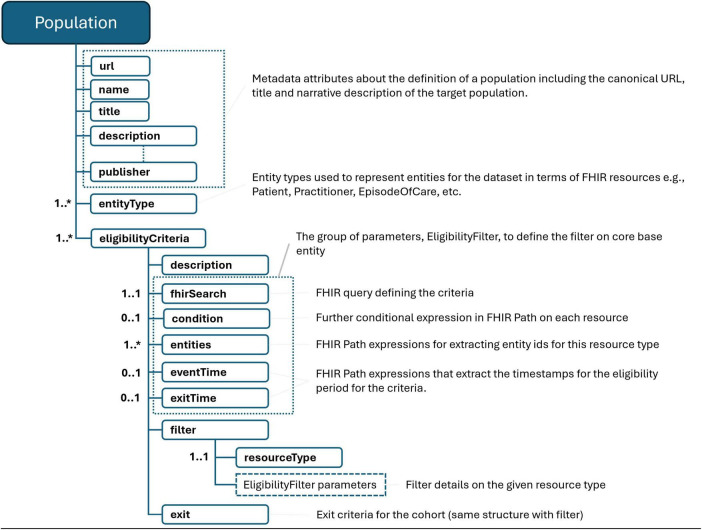
Population definition schema. “*” gives the cardinality of corresponding element and means it is an array and 0 or more cardinality.

As depicted in Figure 1, our methodology employs the names of FHIR resource types, such as “Patient,” to denote that our target population primarily consists of patients, reflecting the common practice in health data analytics. Unlike OHDSI, our approach expands the definition of the target population to include not only individual entities like patients or practitioners but also conceptually broader categories such as encounters or episodes of care. These categories encompass both the individual involved and specific events, such as a hospital visit or a surgical care episode. This broader categorization allows us to leverage the relationships established in FHIR between resources like FHIR Encounter or EpisodeOfCare and other FHIR resources (for example, medications administered during a hospital stay). By doing so, we facilitate precise grouping of data based on distinct criteria, thereby enhancing the clarity and utility of the data for health analytics.

The subsequent step involves defining the eligibility criteria, which detail the characteristics or phenotypes of the entities in question. Our framework supports the definition of multiple eligibility criteria, recognizing that a single entity may exhibit different characteristics based on varying representations of underlying facts. For instance, patients with diminished kidney function might be identified through FHIR Condition resources that implicitly diagnose with specific ICD-10 codes, or through eGFR measurements depicted by FHIR Observation resources where the value falls within a certain range. This flexibility also accommodates the inclusion of various sub-cohorts within the dataset. The process of defining eligibility criteria begins with a FHIR query statement targeting the base entity type, which in our scenario is the FHIR Patient resource. At this example, we impose no limitations on demographic information such as age, gender, or ethnicity, which are typically included in the FHIR Patient resource type. The criteria are further refined through additional filter definitions applied to other FHIR resource types. For example, to isolate patients diagnosed with Parkinson’s disease, we employ a FHIR query on FHIR Condition resources. FHIR facilitates a universal search mechanism via RESTful API, providing a comprehensive list of search parameters for each resource type. These parameters enable queries on FHIR resources using filters based on coded, numeric, Boolean, textual, temporal, or relational information, including references among resources. In our methodology, we utilize these FHIR search statements to delineate a specific result set. In population definitions, these queries serve to filter entities that satisfy at least one condition specified by the query. Specifically, we target patients who have at least one FHIR Condition resource coded with the ICD-10 code “G20” for Parkinson’s diagnosis. Each filter explicitly states the search parameter linking the population to that resource type (e.g., patient = {{Patient}} indicates the ‘patient’ parameter linked to our population’s Patient entities) and includes a FHIRPath expression that specifies the path for entity identifiers (e.g., “Condition.subject”). For more intricate scenarios, additional filters on other resource types can be defined to specify further characteristics required for an entity to be considered eligible for the cohort. Moreover, FHIRPath expressions allow for the imposition of additional conditions on the result set for each filter, addressing constraints that the standard FHIR query mechanism may not accommodate.

In certain cases, entities qualify for inclusion in a cohort only during specific time frames or across multiple intervals. This means that the relevance of an entity to a use case hinges on its state within these designated periods. For instance, in the context of constructing a dataset for analyzing or predicting the progression of Parkinson’s disease, our interest is confined to the period following a patient’s Parkinson diagnosis. By utilizing a FHIRPath expression to mark the event time, we can determine the precise moment each entity enters the cohort, which for our example is the onset time of Parkinson, as recorded in the FHIR Condition resource. While it’s also possible to define an exit time when an entity no longer meets the cohort criteria, this aspect is not utilized in our example scenario. Consider a use case aimed at examining patient outcomes in relation to a specific medication regimen over time. Here, the start and end times of medication administration, as documented in FHIR MedicationRequest resources, could serve as the markers for entering and exiting the cohort, respectively. Given that medication prescriptions are often renewed, multiple resources may document medication use for distinct periods. In such instances, it becomes necessary to identify multiple eligibility periods for patients. Our methodology allows for the specification of a minimum time gap between consecutive eligibility periods. For example, setting a 15-day minimum gap implies that if the interval between two prescriptions is less than 15 days, they are considered part of the same usage period. This approach enables the precise delineation of eligibility periods in alignment with clinical guidelines or practices. As we will detail in forthcoming sections, these eligibility periods—particularly the defined entry and exit times—are critical for the sampling of data used in creating training or validation datasets. They also play a pivotal role in the development of other features that hinge on these specific temporal markers.

The defined entry and exit times within population criteria are instrumental when establishing criteria based on the temporal relationship between two health events. An illustrative scenario, as discussed in the Book of OHDSI, involves identifying “patients who initiate ACE inhibitors monotherapy as first-line treatments for hypertension.” In such a case, one might set up a filter on the Condition resource to search for a hypertension diagnosis, utilizing the diagnosis or onset date as the event time. Subsequently, an additional filter could be applied to MedicationRequest or MedicationStatement resources. This filter would search for a specific set of ATC codes corresponding to ACE inhibitors, incorporating an extra condition. This condition, defined using a FHIRPath expression, would stipulate that the temporal gap between the hypertension diagnosis and the initiation of ACE inhibitor therapy must be at least 365 days. This method enables the precise definition of eligibility criteria that hinge on the chronological sequencing of health-related events.

In addition to inclusion criteria, certain use cases necessitate the establishment of exclusion criteria. Continuing with the aforementioned example, the criterion “with no history of prior hypertension treatment” mandates verifying the absence of any hypertension treatment prior to the identified ACE inhibitors monotherapy, subsequently excluding those patients from the population. This is achieved through the same mechanism of filter definitions, which, when designated as exclusions, allow for the identification and removal of such cases. Entities for which at least one resource meets the FHIR query and the specified condition are thus excluded from the population. This method enables the precise tailoring of the population by omitting entities that do not meet the defined criteria.

#### 2.2.2 Definition of feature groups

**FIGURE 2:**
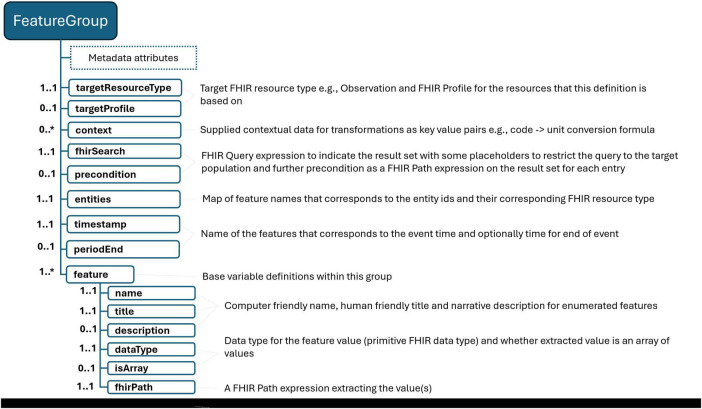
FeatureGroup definition schema. “*” gives the cardinality of corresponding element and means it is an array and 0 or more cardinality.

With an understanding of the necessary features and outcome variables, the following step involves identifying the specific FHIR resources required to compute these variables, ensuring access via the FHIR API while adhering to the agreed-upon common data model. To facilitate the reuse of these definitions across varying scenarios and dataset constructs, we introduce a concept known as a “feature group.” Figure 2 briefly summarizes the definition schema. This construct allows for the delineation of result sets tailored to specific needs. For instance, as depicted in Table 2, one can establish a feature group aimed at gathering blood pressure readings for the targeted population, subsequently isolating systolic and diastolic values as base features for subsequent analyses while calculating other features. Essentially, feature group definitions articulate a FHIR result set—stemming from a particular FHIR query—alongside the specific data points to be extracted from this set.

**TABLE 2:** A sample Feature Group definition to retrieve blood pressure measurements for the specific population.

{
“url”: “https://aiccelerate.eu/feature-groups/pilot1/bloodpressure”,
“name”: “bloodpressure”,
“title”: “Blood Pressure Measurement”,
“description”: “Represent a blood pressure measurement including systolic and diastolic”,
“version”: “0.1”,
“date”: “2022-09-07”,
“fhirVersion”: “4.0.1”,
“publisher”: “AICCELERATE WP1 Team (SRDC Corp.)”,
“targetResourceType”: “Observation”,
“targetProfile”: “http://hl7.org/fhir/StructureDefinition/bp”,
“fhirSearch”:
”?patient = {{Patient}}&category = http://terminology.hl7.org/CodeSystem/observationcategory{nT1ntextbar} vital-signs&code = http://loinc.org\T1\textbar} 85354-9”,
”entities”: {
“pid”: “Patient”
},
“timestamp”: “time”,
“feature”: [
{
“name”: “pid”,
“title”: “Patient identifier”,
“description”: “Patient identifier”,
“dataType”: “id”,
“fhirPath”: “Observation.subject”
}, {
“name”: “time”,
“title”: “Observation time”,
“description”: “Time of measurement”,
“dataType”: “dateTime”,
“fhirPath”: “Observation.effectiveDateTime”
},
{
“name”: “systolic”,
“title”: “Systolic BP”,
“description”: “Systolic BP value”,
“dataType”: “decimal”,
“fhirPath”:
“Observation.component.where(code.coding.exists (system = ‘http://loinc.org’ and code = ‘8480-6’)).first().valueQuantity.value”
},
{
“name”: “diastolic”,
“title”: “Diastolic BP”,
“description”: “Diastolic BP value”,
“dataType”: “decimal”,
”fhirPath”: “Observation.component.where(code.coding.exists (system = ‘http://loinc.org’ and code = ‘8462-4’)).first().valueQuantity.value”
}
]
}

Similar to defining a population, we employ FHIR search statements to outline the desired result set, specifying both the type of FHIR resource and the expected target FHIR profile to ensure the resulting resources conform accordingly. In the given example, we opt for the FHIR Blood Pressure profile, which mandates the use of the LOINC code 85354-9 to identify blood pressure measurement records specifically. This code is utilized as a filter within the search statement. Additionally, the relevant FHIR reference or ID type search parameter is paired with an entity placeholder (e.g., patient = {{Patient}}). This approach signifies that our request is exclusively for records pertaining to patients within the defined population, ensuring that the data collected is directly relevant to our study’s subjects.

We proceed to identify the base variables to be extracted from the result set determined by the FHIR search statements. This compilation should encompass potential identifiers for the entities involved, and, where applicable, associated time information that elucidates the timing of the clinical event or fact in question. FHIR resources, akin to other clinical record models, are capable of representing health-related facts or events in three distinct categories: (i) time-independent information, such as demographics provided by the FHIR Patient resource or family health conditions outlined in the FHIR FamilyMemberHistory resource; (ii) events/facts associated with a specific time point, like the onset date of a chronic condition detailed in the FHIR Condition resource or a particular laboratory result specified in the FHIR Observation resource; and (iii) events/facts pertinent to a defined time period, for instance, the duration of medication use indicated by the FHIR MedicationStatement resource. In the construction of these definitions, it is crucial to map entity identifiers to their corresponding entity types. For instance, in our scenario, we designate “pid” as the identifier for patients. Additionally, temporal variables—such as the timestamp of the clinical event/fact or the start and end times for events/facts spanning a period—must be articulated for the feature groups, except those involving time-independent information. In our case, we specify that the variable “time,” representing the moment of blood pressure observation, will serve as the timestamp for the data in question.

Illustrated by our example, each variable is accompanied by metadata including its name, description, and data type (aligned with FHIR data types), along with a FHIR Path expression that specifies the method for extracting information from the result set. Beyond mere extraction, FHIR Path can be employed for data transformations or calculations. For instance, in situations where your common data model does not limit the units for a particular laboratory result or if there are several unit options, FHIR Path expressions can be used to convert numeric values from various units into a standardized unit, facilitating data harmonization. Similarly, these expressions can be applied to rescale or discretize numeric values, aiding in data normalization. Our approach allows for the inclusion of such contextual data within the definition itself, providing formulas for unit conversion, thresholds for clinical measurements, etc. This enables the use of FHIR Path expressions for performing the requisite calculations. By integrating contextual data and its metadata within the dataset definition and keeping it separate from the scripts, we adhere to our principles of transparency and readability. Additionally, this method enhances the configurability and reusability of the definitions.

#### 2.2.3 Definition of dataset

**FIGURE 3:**
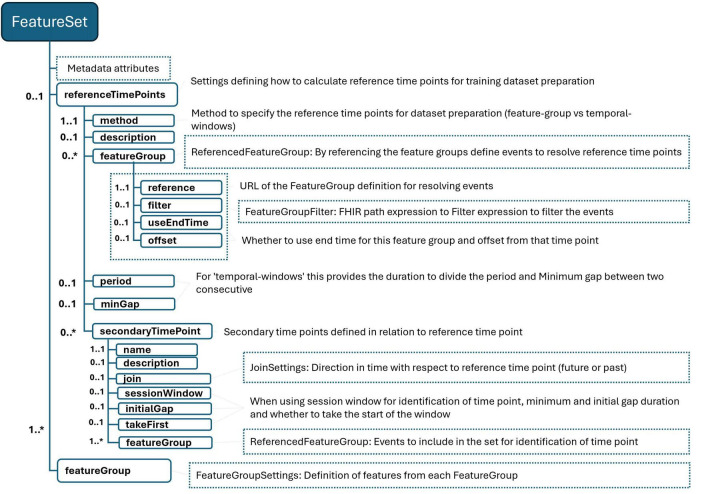
FeatureSet definition schema. “*” gives the cardinality of corresponding element and means it is an array and 0 or more cardinality.

**FIGURE 4:**
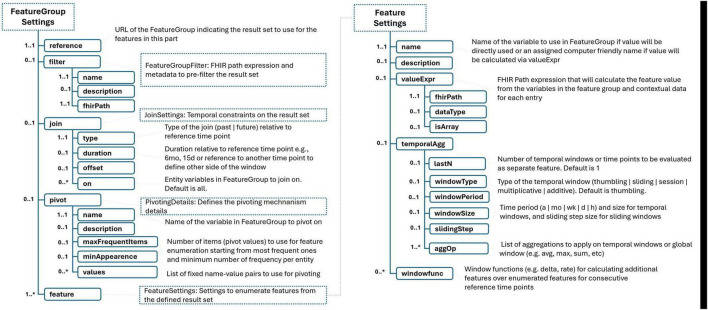
Remaining of FeatureSet schema. “*” gives the cardinality of corresponding element and means it is an array and 0 or more cardinality.

We introduce the concept of “feature set”; similar to other constructs within our framework, its definition begins with essential metadata that provides a verbal description of the dataset to be prepared with respect to the feature set definition. The definition model is illustrated in Figures 3, 4. We outline a strategy for resampling the longitudinal health data, which often displays characteristics of sparsity and irregular sampling intervals, with various variables being recorded at disparate frequencies. The pivotal decision here involves determining the sampling time points for each entity, essentially deciding what each row in the dataset represents. This decision is intricately linked to the specific analytics or AI use case envisioned for the dataset. Current practices in the literature, employed by data scientists and researchers, offer several approaches for this:

• Selecting the start or end times of specific health events as sampling points. For example, utilizing the discharge time from a hospital as the sampling point for a dataset aimed at predicting hospital readmission.
• Segmenting a period to establish sampling points based on the frequency of the most regularly recorded data. An instance of this would be dividing the time from the end of surgery until discharge into 8-h intervals for a dataset intended to predict the length of stay following cardiac surgeries.
• Dividing a period while also incorporating outcome events into consideration. For example, segmenting the duration of an Intensive Care Unit (ICU) stay into 5-min intervals, but also using the occurrence time of sepsis as an additional sampling point and adjusting the time windows accordingly. This approach aims to predict sepsis during ICU stays by analyzing vital signs and other frequent measurements, ensuring snapshots of each patient are taken at 5, 10, 15 min, etc., prior to the observation of sepsis.

These strategies enable the creation of datasets that reflect the dynamics of patient health status over time, tailored to the specific analytical or predictive needs of the use case.

Within our framework, we’ve integrated a mechanism to streamline the definition of sampling strategies, as illustrated in Table 3 under the “referenceTimePoints” section. This mechanism allows users to specify the methodology for determining sampling time points in a structured manner:

• Method: The “method” element specifies the chosen methodology for sampling. For methods that require dividing a period into sub-periods, we leverage the eligibility period calculated for each entity based on the population definition. For instance, in a scenario where a patient’s eligibility period is delineated by the time span from the end of their first surgery to their discharge from the hospital, specifying a period (e.g., 1 h) means this duration will be segmented into 1-h intervals.
• Outcome Events: If the determination of time points also takes into account certain outcome events, these are specified by referencing one or more FeatureGroup definitions. In the given example, a FeatureGroup that provides data on complications is utilized for this purpose.
• Configuration: Users can further refine the strategy by setting a time offset to define the exact sampling point relative to an event, as well as a minimum gap between two outcome events for them to be considered distinct outcomes. In the context of predicting post-operative complications, the example specifies that two complications must occur at least 8 h apart. Additionally, it stipulates that the initial sampling point should be set 1 h before the occurrence of the earliest complication.

**TABLE 3:** A sample FeatureSet definition−Defining sampling time points and other time points.

{
“url”: “https://aiccelerate.eu/feature-sets/pilot1_hsjd_complications”,
“name”: “pilot1_hsjd_complications”, “title”: “Feature set for AICCELERATE Pilot 1 for predicting complications after the surgery”,
“description”: “Feature set for AICCELERATE Pilot 1 for predicting complications after the surgery”,
“version”: “0.1”,
“date”: “2022-04-21”,
“fhirVersion”: “4.0.1”,
“publisher”: “AICCELERATE WP1 Team (SRDC Corp.)”,
“referenceTimePoints”: {
“method”: “temporal-windows”,
“description”: “The time period between the end of first surgical operation and the discharge time is divided into 1h periods. However if patient has complications then these event times are considered as anchor points and reference time points are calculated accordingly. Two complications with less than 8 h are assumed same complication so no reference time point is picked within this period. Enumeration for reference time points start from 1 h before the complications”,
“useEndTime”: true,
“period”: “1h”,
“minGap”: “8h”,
“offset”: “1h”,
“featureGroup”: [{
“reference”:”https://aiccelerate.eu/feature-groups/pilot1/complication”
}],
“secondaryTimePoint”: [
{
“name”: “lastSurgeryTime”,
“description”: “Time of the latest main surgery performed in the episode”,
“join”: { “type”: “past” },
“featureGroup”: [
{
“reference”:”https://aiccelerate.eu/feature-groups/pilot1/surgeryEncounter”,
“useEndTime”: true,
“filter”: {
“name”: “isMainSurgery”,
“description”: “If procedure is main cardiac surgery”,
“fhirPath”: “category = ‘394603008’”
}
}
]}
]},
……

This flexible mechanism enables precise configuration of sampling strategies, tailoring the dataset to capture clinically relevant events and periods. By incorporating both fixed intervals and event-driven sampling points, researchers can create datasets that more accurately reflect the complexities of patient care trajectories, enhancing the potential for insightful analysis and predictive modeling.

In addition to primary sampling points, certain scenarios benefit from the delineation of secondary time points, which correspond to significant health events within the patient’s care continuum. These secondary time points are defined in relation to the primary sampling points, offering a nuanced timeline that captures critical clinical milestones. In the provided example of Table 3, the secondary time point “lastSurgeryTime” is identified as the time marking the end of the patient’s last surgery, as indicated by the relevant feature group that records surgery encounters. This point is determined to be the closest, yet prior, instance to the established primary sampling time point. To ensure the significance of each identified event, users have the flexibility to specify a minimum interval that should exist between two consecutive events. Furthermore, the framework allows users to select specific events (e.g., first, last, second to last) to serve as these secondary time points. Secondary time points, along with primary sampling points, play a crucial role in defining the temporal context for data analysis and feature extraction. For instance, in the example, the “lastSurgeryTime” serves as a pivotal reference for calculating features such as the number of hours elapsed since the most recent surgery at each sampling point. This approach allows for the inclusion of dynamic, temporally relevant information in the dataset, enhancing the precision of subsequent analyses and the development of predictive models that accurately reflect patient trajectories and outcomes.

The process of transforming raw health data into meaningful dataset features involves defining a set of temporal and contextual constraints, aggregations, and transformations based on the information provided by related feature groups. Each feature group encapsulates a category of health events (e.g., lab results, diagnoses, surgeries) along with the base facts of these events, the entity they are related to, and the time or period of the event. To convert these facts into actionable variables, it’s essential to establish clear temporal relationships between the facts represented by the selected feature groups and the predefined anchor time points. For instance, as illustrated in Table 4, when defining features, one may only want to consider medication usage data from the most recent three months. This decision impacts the definitions of features within the dataset, such as a Boolean feature indicating recent benzodiazepine use by a patient. This “recency” is calculated in relation to the main sampling time point for each record, ensuring that the feature reflects current or recent medication use. The language designed for this purpose allows users to define temporal constraints with ease, specifying periods in relation to defined time points either by indicating a duration that looks forward (future) or backward (past) in time. This flexibility can include optional offsets or can be bounded between two specific time points. For example, to focus on diagnoses made after a patient’s Parkinson’s diagnosis, one could define a temporal period that spans from the time of the patient’s eligibility to the sampling time point. This approach facilitates the generation of features that are not only relevant to the patient’s current health state but also temporally aligned with the objectives of the study or analysis. It allows for the creation of datasets that can more accurately model health outcomes by incorporating the timing and sequence of health events in relation to significant clinical milestones.

**TABLE 4:** A part of sample FeatureSet definition – Defining features from medication data for predicting progression to Advanced Parkinson Disease.

{
“reference”:”https://aiccelerate.eu/feature-groups/pilot2/medication”,
“join”: {
“type”: “past”,
“duration”: “3mo”
},
“feature”: [
{
“name”: “hasBenzodiazepinesRecently”,
“description”: “Whether patient has benzodiazepines or not within this period. ATC Code: under N05CD”,
“valueExpr”: {
“fhirPath”: “atcCode.startsWith(’N05CD’)”,
”dataType”: “boolean”
},
”temporalAgg”: [{
“aggOp”: [”any”]
}]
},
…

In the process of defining features for a dataset, it’s not only possible to apply temporal constraints to health event data, but you can also impose contextual constraints to further refine the data included in your analysis. This is accomplished by specifying filters on the data represented by a feature group. These filters are expressed using FHIRPath expressions, which allow for precise selection of data based on specific criteria. Table 5 shows an example of such a contextual constraint filtering complication data, where the result set is based on FHIR AdverseEvent resources. By applying a filter using the corresponding SNOMED-CT code, one can specifically target unexpected ICU admission events. This method ensures that the dataset only includes relevant adverse events, thereby enhancing the specificity and relevance of the analysis.

**TABLE 5:** A part of sample FeatureSet definition−Enumerating features from frequent SPO2 measurements in hospital after cardiac surgeries for predicting complications.

{
“reference”: “https://aiccelerate.eu/feature-groups/pilot1/vitalsign”,
“filter”: {
“name”: “spo2”,
“fhirPath”: “code = ‘2708-6’”
},
“feature”: [
{
“name”: “value”,
“description”: “Aggregation of last (2,4 and 8)-h time windows for SPO2 measurements”,
“temporalAgg”: [
{
“lastN”: 3,
“windowPeriod”: “h”,
“windowSize”: 2,
“extending”: “multiplicative”,
“aggOp”: [”stddev”, “avg”, “max”, “min”, “median”, “kurtosis”, “skewness”]
}
],
“windowFunc”: [”delta”]
},
{
“name”: “value”,
“description”: “Aggregations of last 3 1-h time windows for body SPO2 measurements”,
“temporalAgg”: [
{
“lastN”: 3,
“windowPeriod”: “h”,
“windowSize”: 1,
“aggOp”: [”stddev”, “avg”, “max”, “min”, “median”, “kurtosis”, “skewness”]
}
],
“windowFunc”: [”delta”]
}
]
},
…

The language introduces a “pivoting mechanism” for efficiently handling scenarios where it’s necessary to generate a standardized set of features across multiple concepts within the same category, such as laboratory test results. This mechanism is particularly useful for cases where analysts wish to extract a common suite of statistical measures (e.g., the latest, average, minimum, and maximum values) for a variety of tests or measurements that are relevant to their specific use case. The first step involves selecting a base variable from the FeatureGroup definition that will serve as the pivot. This could be, for example, the LOINC code for a laboratory test, which uniquely identifies the type of lab test being conducted. Users can then specify a list of values and corresponding labels for this pivot variable. These values could be specific LOINC codes for lab tests that are of particular interest in the use case. If the exact tests of interest are known ahead of time, they can be explicitly listed in the model. If the specific items of interest are not predetermined, the model allows users to define criteria for automatically selecting these pivot variables based on the data. For instance, one might specify that features should be enumerated for the 20 most frequently occurring lab tests in the dataset, provided that each of these tests appears in the records of at least 100 patients. This pivoting mechanism simplifies the process of generating a consistent set of features across multiple data points or concepts, which is particularly valuable when dealing with large and complex datasets. It ensures that analysts can focus on analyzing the most relevant and frequently occurring data points without manually defining features for each possible variable.

In the process of defining features for a dataset, there are two primary methods to derive feature values from the underlying data represented by feature groups: direct use of base variable values and calculation through FHIR Path expressions.

• Direct Use of Base Variables: A feature can be directly based on the value of a base variable that has been defined within the related feature group. This approach is straightforward and involves using the raw value of a data point as a feature in the dataset. Table 5 shows an example using the “value” variable from a feature group that represents vital sign information.
• Calculation Through FHIRPath Expressions: Alternatively, features can be derived by applying FHIRPath expressions to calculate values from the data records within each feature group. This method allows for more complex transformations of the data. As shown in Table 4, an example illustrates how medication usage data, identified by ATC codes in the medication usage feature group, can be transformed into a feature indicating whether the patient is using a medication from the benzodiazepine group. This involves interpreting the ATC codes using FHIRPath expressions to identify specific medication classes and then summarizing this information into a binary feature (e.g., benzodiazepine usage: yes/no).

For addressing the challenges of data harmonization, especially when dealing with disparate measurement units, scales, or categorization needs stemming from different calibration standards of medical devices or varied clinical practices, the model introduces a mechanism for specifying and applying contextual information. The model facilitates this through a dedicated section within FeatureGroup or FeatureSet definitions, designed for the transparent declaration of contextual parameters. These parameters can encompass a wide array of transformational instructions, such as:

• Conversion formulas for standardizing units of measurement (e.g., converting temperature from Fahrenheit to Celsius or blood pressure readings from mmHg to kPa).
• Rescaling instructions for numerical values to align with a common scale or range, enhancing comparability.
• Categorization criteria based on clinical thresholds or norms, enabling the transformation of continuous data into discrete categories that reflect clinical significance (e.g., defining hypertension stages based on blood pressure readings).
• Terminology mappings, which are crucial for harmonizing data coded in different clinical terminologies or classification systems, such as mapping between different coding systems for diagnoses or medications (e.g., ICD to SNOMED-CT).

The proposed language provides a convenient method for generating multiple features from a single value by leveraging a combination of aggregation operators and temporal windowing strategies. This approach allows for the extraction of rich, time-sensitive insights from health data, particularly useful for variables that are measured repeatedly over time, such as vital signs or lab results. The key aspects of this feature include:

• Aggregation Operators: Users can apply a variety of standard aggregation functions, such as standard deviation, average, and maximum, to a set of data points. These functions are akin to those found in SQL and data processing frameworks like Apache Spark (20), ensuring familiarity and ease of use for those with a background in data science.
• Temporal Windowing: The language supports several types of temporal windows, including tumbling, extending, session, and sliding windows. This flexibility allows users to analyze data over specified periods in a manner that best suits their analytical or predictive needs. For example, users can look at the last 3 1-h windows or extend their analysis over longer periods, such as 2, 4, and 8 h, to observe trends or changes over time.
• Configuration Flexibility: Parameters such as the number of windows, window size, extension factor, or sliding step duration can be easily adjusted. This configurability enables users to tailor their analysis to specific requirements or hypotheses about the data.
• Extension Capability: While the language comes with a set of predefined aggregation operators, it is designed to be extensible. Implementors can introduce additional operators as needed, enhancing the language’s applicability to a wide range of scenarios and datasets.
• Delta and Rate of Change: Beyond simple aggregations, the language supports operators for calculating changes between consecutive temporal windows, such as the delta or rate of change. This feature can be particularly insightful for tracking the progression or improvement of a patient’s condition over time, offering a dynamic view of health status that static measurements cannot provide.

**FIGURE 5:**
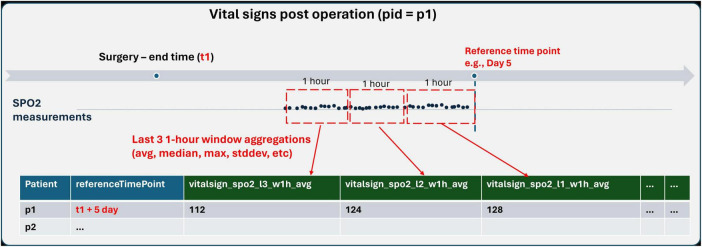
Population definition schema.

As exemplified in Table 5 and Figure 5, by applying these techniques to SpO2 (oxygen saturation) measurements, users can generate a comprehensive set of features that describe not just the current state but also the variability and trends of a patient’s oxygen levels over time. Such detailed feature sets can significantly enhance the predictive power of analytical models, enabling more nuanced and accurate assessments of patient health and outcomes.

We introduce a systematic naming convention within the language to ensure that each feature generated through its advanced aggregation and temporal windowing capabilities receives a unique and descriptive name. This naming scheme is crucial for maintaining clarity and ease of reference when dealing with a potentially large number of features. The components of this naming scheme include:

• FeatureGroup Names: The base name derived from the FeatureGroup, which categorizes the health event or data type, e.g., “vitalsign”.
• Filters: The specific aspect or measurement within the FeatureGroup, such as “spo2” for oxygen saturation levels.
• Join Expression and Temporal Aggregation Window: Indicators such as “l2” and “w1h” specify the temporal context of the feature, with “l2” denoting the second last window and “w1h” specifying a window period of 1 h.
• Aggregation and Window Function Operators: The operation applied to the data, such as “avg” for average, clearly indicates the type of statistical measure calculated for the feature.

As illustrated in Figure 5, “vitalsign_spo2_l2_w1h_avg”, illustrates how these elements combine to form a feature name that is both informative and concise. This feature name indicates that it represents the average oxygen saturation (“spo2”) values from the “vitalsign” feature group, calculated over the second last 1-h window (“l2_w1h”). This structured approach to naming ensures that each feature’s purpose and derivation are immediately apparent, facilitating easier analysis and interpretation of the data. It also aids in the automated processing of features, as the naming convention provides clear and consistent cues about the nature and temporal dynamics of the data encapsulated by each feature.

## 3 Results

### 3.1 Implementation: a feature repository for health data

We have developed a software, onfhir-feast, capable of processing declarative data preparation pipeline definitions in a high-performance distributed manner. This software enables two key functionalities: (1) Batch extraction of training or validation datasets from an integrated FHIR compliant data source and (2) Calculation of features for entities (e.g., patients) to support online prediction services integrated into the production environment as part of an AI-based decision support solution.

onfhir-feast is aligned with the emerging concept of a feature store, which is integral to AI pipelines. In the realm of machine learning, a feature store serves as a platform dedicated to managing and providing access to both historical and real-time feature data (21). It facilitates the creation of precise datasets at particular time points using historical feature data. Consistent with this definition, onfhir-feast manages Population, FeatureGroup, and FeatureSet definitions to provide a REST API for configuring or triggering a dataset extraction pipeline, enabling access to the dataset or real-time features for online predictions by leveraging these definitions.

A service built on an EHR system that provides data access via HL7 FHIR is typically optimized for patient-centric applications with user interfaces. However, when it comes to population-centric queries, especially in the context of AI pipelines, performance issues may arise due to the large volume of data involved. To address this challenge and prevent excessive workload on FHIR endpoints, onfhir-feast is designed akin to a health data warehouse. In this setup, only relevant data is synchronized periodically, typically at intervals such as every hour or every day, based on the Population and FeatureGroup definitions in the platform. Consequently, only FHIR resources updated since the last synchronization will be queried, resulting in a reduced workload on the system ensuring optimal performance and efficient utilization of resources.

In this synchronization process, the Population definitions take precedence. Entity identifiers of the resulting entities identified in each batch, based on these Population definitions, are stored in a specific population table within the configured time-series data repository. Subsequently, each FeatureGroup definition referenced in the activated dataset definitions is executed for the related population identified up to that point in time. This approach ensures that the platform only synchronizes the necessary data for the identified population.

The result sets of FeatureGroup executions are likewise stored in FeatureGroup-specific tables in the time-series data repository. Importantly, Population and FeatureGroup definitions can be reused across different dataset definitions. The platform manages this seamlessly to ensure that it never queries and processes the same FHIR resource more than once, thereby optimizing efficiency and resource utilization.

With this synchronization mechanism, onfhir-feast acts as a data warehouse similar to having an OMOP database populated with data pipelines mapping EHR system data. But in our case, users are more flexible to design their own tables, in other words Feature Groups, tailored to their use cases when needed.

Additionally, onfhir-feast offers an API to asynchronously trigger dataset extraction for preparing training or validation datasets. Users can choose to utilize all available data or specify a particular period, such as extracting a training dataset from data recorded in the previous year. Similarly, periodic dataset preparations can be scheduled and configured to support AI model retraining scenarios. When such an extraction is triggered, the platform initiates the synchronization phase, which updates with the new data on the integrated system until the last synchronization point and populates the related tables in the time-series data repository. Subsequently, the relevant FeatureSet definition is executed on the loaded data from those tables for the identified entities within the population to prepare the dataset. The resulting datasets are then stored in the integrated “Offline Feature Repository.”

Throughout the execution of this process, the client has the option to inquire about the status of the process via the REST API. Upon completion, automatically generated metadata of the dataset is stored and made accessible to the client. This metadata includes a list of features and outcome variables, along with their descriptions, basic statistics (such as the number of missing values, maximum, minimum, and average for numeric values, cardinalities, and value sets for nominal features, etc.).

The platform leverages Apache Spark (20), Akka (22) and Apache Software Foundation (23) frameworks to ensure reliable, fault-tolerant distributed processing for handling parallel FHIR queries during population identification and synchronization phases, as well as processing the result set for dataset preparation. It also provides extension mechanism to support the usage of different type of databases or persistency mechanisms as integrated repositories (such as time series data repository, Offline and Online Repository). Currently, PostgreSQL based repositories and file system-based repositories for storing data in Apache Parquet format are supported.

The platform can also serve as a component of a decision support application integrated with a trained AI model, tasked with preparing features for individual entities for online prediction. To facilitate this, a corresponding synchronous operation is provided as part of the REST API. During this process, the same dataset definitions, comprising the bundle of FeatureSet, Population, and FeatureGroup definitions, are executed. However, this time, they are applied to a single entity (e.g., a patient) to calculate the same feature list in a consistent manner. The process triggers the synchronization phase solely for that entity, considering data updated after the last synchronization time for the target population if the patient is included in the population. Subsequently, the FeatureSet definition is executed on the obtained data to prepare the features for online prediction.

### 3.2 Case studies

The proposed methodology and the implemented platform have been deployed and tested in 3 research projects supported within the EU Horizon 2020 and Horizon Europe frameworks namely, AICCELERATE (24), DataTools4Heart (25) and AI4HF (26) projects as part of several pilot studies.

Table 6 presents the list of pilot studies and use cases where the described methodology is followed and a range of dataset definitions are provided. In all these projects, the onfhir-feast platform is deployed locally on the data provider’s data center to extract training and/or test datasets. For instance, in AICCELERATE, Pilot 2 involves utilizing datasets extracted from various data providers for cross-validation of AI models developed locally. Additionally, onfhir-feast serves as an integral component of the resulting solution for online prediction. In DataTools4Heart and AI4HF projects, onfhir-feast is incorporated into federated learning platforms to extract harmonized datasets from diverse data providers.

**TABLE 6:** Case studies where the methodology and the platform are used for preparing training/validation datasets.

Case study	# of data provider	# of variables
**AICCELERATE**		
Pilot 1−Patient Flow Management and Surgical Units	2	
1.1 Dataset for predicting complications after cardiac surgeries	1	916
1.2 Dataset for predicting length of stay (LoS) for patients after cardiac surgeries	1	583
1.3 Dataset for predicting duration of surgery, ICU stay and LoS before surgery	2	88
Pilot 2−Parkinson’s Disease Digital Care Pathway	2	
2.1 Dataset for predicting progression to advanced Parkinson stage and predicting Mild Cognitive Impairment in Parkinson patients	2	402
Pilot 3−Palliative and chronic pediatric service delivery & patient workflow	3	
3.1 Dataset for clustering pediatric palliative patients into risk groups	1	117
3.2 Dataset for predicting tumor relapse after cancer treatment in pediatric patients	1	549
3.3. Dataset for predicting time needed for preparation (time to surgery) to a surgery	1	48
DataTools4Heart and AI4HF*		
Pilot 1−Medication prescription in patients with acute heart failure and chronic kidney disease or hyperkalaemia	9	
1.1 Dataset for analysing prescription patterns and clinical outcomes in terms of HF and CKD	9	604
Pilot 2−Risk score for acute HF in the emergency department	9	
2.1 Dataset for predicting on (HF/CV)-rehospitalization, cardiovascular event or mortality within 7-, 30-, 90-, 180-days, 1-, 3- and 5-year follow-up.	9	162
Pilot 3−Referral pathways for patients with HF	9	
3.1 Dataset for predicting the right specialty at the first time right to refer the patients for an in-hospital and general practitioners referral support model.	9	268

*DataTools4Heart and AI4HF projects are under development at the time of writing this manuscript. The numbers might change as the projects may evolve.

#### 3.2.1 Example case study−predicting complications after cardiac surgeries

To illustrate the methodology and results achieved using the solution, we will now provide the details of the data preparation pipeline for one of the use cases within the AICCELERATE project’s pilot 1 study. This particular use case revolves around predicting complications, specifically unexpected ICU admissions following cardiac surgeries and specific diagnostic procedures.

For this study, the target cohort is defined as the surgical episodes of patients who have undergone at least one cardiothoracic surgery or diagnostic procedure, such as cardiac catheterization or cardiac electrophysiology. These eligibility criteria are defined using a Population definition, which filters the FHIR EpisodeOfCare and Encounter resources based on the service type of encounter, utilizing the corresponding SNOMED-CT codes.

Within these episodes of care, which encompass the period from hospital admission to discharge, various types of encounters occur, including surgical encounters, ward stays, intensive care unit (ICU) stays, and pre-surgery visits. The study utilizes diagnostic data and basic patient demographic information from the pre-surgery phase. Additionally, it incorporates details of surgical or diagnostic procedures performed, including specific interventions such as intubation, defibrillation, and hypothermic circulatory arrest, which may influence post-operative complications. Furthermore, intraoperative observations and assessments, such as minimum temperature and related surgery risk scores, are included in the study. For the post-operative phase, a specific set of lab results and frequent vital sign measurements obtained during ICU or ward stays are primarily utilized for prediction purposes. For instance, the data provider’s dataset includes vital signs recorded at 5-min intervals for most of the time until discharge. To calculate outcome variables, a list of explicit complication data, including a range of post-operative complications and unexpected ICU admission events, is employed.

Table 7 illustrates the HL7 FHIR-based common data model and feature group definitions provided for the use case, along with their relationships. For instance, the FeatureGroup definitions “icuOrWardStay” and “surgeryEncounter” are dependent on the model described by AIC-OperationEpisodeEncounter, which customizes the FHIR Encounter resource model. On the other hand, the definition named “surgeries” relies on two profiles: one customizing the record representing the main surgical procedure and the other representing additional procedures performed in relation to cardiac surgeries. The table also details the primary customizations or restrictions applied to the standard resource model for each defined profile, as well as the parameters extracted from those records within the FeatureGroup definitions.

**TABLE 7:** Feature group definitions and relation to CDM for the use case.

FHIR resource type	AICCELERATE CDM FHIR profile	Feature group definitions
Patient	AIC-Patient: Patient demographics - gender, birthdate → Set as mandatory	Patient_demographics (pid, gender, birthDate)
EpisodeOf Care	AIC-OperationEpisode: Surgical episode of care indicating the period from admission to discharge - type → Bind to a valueset for episode types to distinguish surgical episodes - diagnosis → Set as mandatory to identify pre-operative diagnosis for surgery	Episodes (pid, episodeId, time, endTime, preOpDiagnoses, comorbidtyDiagnoses)
Encounter	AIC-OperationEpisodeEncounter: Encounters related to surgical workflow. - type → Bind to a ValueSet with SNOMED-CT codes to distinguish ICU stays, ward stays, operation encounters	Icuorwardstay (pid, episodeId, encounterId, startTime, endTime, type, location, duration)
		surgeryEncounter (pid, episodeId, encounterId, startTime, endTime, category, priority, location, duration)
Condition	AIC-Condition: Diagnosis records for patients - code → Bind to ICD-10-CM value set	Condition (pid, encounterId, onsetDate, icd10Code)
Procedure	AIC-SurgeryPhaseDetails: Record to provide details of the main procedure performed in surgery. - category → Identify a fixed SNOMED-CT code to distinguish such records - code → Bind to ICD-10-PCS value set for surgery codes	Surgeries: Details of the surgery (pid, episodeId, encounterId, startTime, endTime, isMainSurgery, ccsCategory, mainProcedureCode, bodySite, duration, aristotleScore, stsScore, rachs1Score, extubationStatus, defibrillationStatus, minTemparature, cecTime, clampTime, arrestTime)
	AIC-ProcedureRelatedWithSurgicalWorkflow: Other related procedures performed in surgery−code → Bind to a ValueSet for interested procedure codes in SNOMED-CT for cardiac surgeries e.g. extracorporeal circulation procedure (cec), vascular clamp, extubating, defibrillation, etc.	
Medication Administration	AIC-MedicationAdministration: Record indicating an administered medication within surgical workflow in the hospital. - medication → Bind to ATC codes	Medications (pid, episodeId, time, atcCode, atcCategory, dose, doseUnit)
Observation	AIC-LabResultWithinSurgicalWorkflow: Record providing a related lab result - code → Bind to LOINC codes for lab results and provide a ValueSet to declare the interested lab tests for the use case	Lab (pid, episodeId, encounterId, time, code, value, unit, interpretation)
	Vitalsigns: A set FHIR standard profiles representing vital sign measurements e.g. body weight, temperature, SPO2, blood pressure, etc. - Fixed LOINC codes and units for each vital sign	Vitalsign (pid, time, code, value)
		bloodpressure (pid, time, systolic, diastolic)
AdverseEvent	AIC-ComplicationAfterOperation: Record indicating an adverse event after surgical operation. - event → Bind to a ValueSet including SNOMED-CT codes listing interested complications occur after cardiac surgeries including unexpected ICU admission	Complication (pid, episodeId, encounterId, time, code)

List of features and outcome variables that are designed for this use case in collaboration with clinicians and data scientists are provided in supplementary material as Supplementary Table 1. Related definitions are available open source at https://github.com/aiccelerate/data-extraction-suite/blob/main/definitions/pilot1-hsjd/.

Within this pilot study, the data is provided by the project partner Sant Joan de Deu hospital by getting data exports from corresponding EHR database tables in CSV format. For the transformation of data in CSV files into HL7 FHIR resources, the open source toFHIR platform (27, 28) is used as data integration platform, and onFHIR.io (29) is utilized as the secure health data repository.

The onfhir-feast tool, along with the data integration platform, is deployed on a server for demonstration and piloting purposes. Utilizing the toFHIR tool and corresponding mapping definitions, retrospective data provided in CSV format are transformed into FHIR resources compatible with the CDM for the specified use case. These FHIR resources are then stored in the onFHIR.io repository. Table 8 provides an overview of the data size, indicating the number of FHIR resources created as a result of the mappings. Following this, a batch dataset extraction job is initiated on onfhir-feast using the designed dataset preparation pipeline definition to create the dataset for training and testing of AI models. Moreover, the setup serves as an integral part of the prediction service served to healthcare professionals wrapping the trained AI model. The prediction service and UI component utilize onfhir-feast APIs to retrieve features for a patient within a surgical episode. Subsequently, this information is utilized for online prediction of complications for that patient.

**TABLE 8:** Number of FHIR resources created by mapping raw data and used in dataset creation.

FHIR resource	# of relevant resources	Details
Patient	906	
Episode of Care	1,022	
Encounter	4,581	Surgical encounters: 1,197 ICU stays: 783 Ward stays: 1294
Condition	2,310	
Medication administration	121,188	
Procedure	2,210	1,197 surgery 1,013 other procedures records
Observation	6,972,703	6,853,917 vital sign records 76,191 lab result records 1,108 others 41,487 blood pressure records
Adverse event	565	

We conducted a basic performance test using the same setup on a single personal computer (Lenovo ThinkPad) equipped with an 11th Gen Intel(R) Core (TM) i7-11800H processor running at 2.30GHz. The test was carried out within a controlled Docker environment featuring 8 CPU cores and 16GB of RAM. Initially, we executed the synchronization job independently, as the synchronization phase relies on the performance of the FHIR server to respond to queries. Subsequently, the dataset preparation job was performed, taking approximately 164 min to complete. The resulting dataset comprises 916 variables and 141,805 entries, covering 1,022 surgical episodes belonging to 906 patients. Furthermore, the metadata generation for this dataset, including basic statistics, required approximately 2 min. The API for retrieving features for a patient within a surgical episode at any chosen time demonstrated an average response time of 37 s.

## 4 Discussion

### 4.1 Principal findings

In this paper, we have introduced a declarative data preparation pipeline definition language designed to transparently outline each stage of the transformation process from EHR data to AI-ready feature sets. This framework ensures traceability by providing a clear depiction of the transformation and pre-processing operations applied to the data, from its retrieval from EHRs to its delivery to AI models for training.

Through our implementation in our pilot studies, we have demonstrated that, the framework is extensive enough for defining diverse set of features with different temporal and contextual criteria. In the realm of applying machine learning to electronic health record (EHR) data, researchers frequently resort to readily extracted, manually chosen obvious features due to the time-intensive nature of more thorough preprocessing methods (6). The proposed dataset definition language enables researchers to easily enumerate features with different representations and temporal context using FHIR Path expressions, temporal windows, aggregation operators and window functions. Furthermore, as the important part of the definitions are parametrized, users have the chance to generate different versions of the datasets with different configurations which helps them to search for optimal or suitable solution with the underlying data. This capability is invaluable for researchers seeking the most effective or appropriate analytical models based on the available data, enabling a more dynamic and exploratory approach to data analysis.

Reproducibility poses a common challenge in AI research, with healthcare presenting a particularly pronounced instance of this issue. The limited availability of publicly accessible medical datasets serves as one indication of this challenge (30). While promoting increased data sharing is crucial, establishing reusable and standardized definitions for key concepts such as target cohorts, phenotypes, and datasets, as advocated in this article, can significantly enhance reproducibility in AI research. Encouraging researchers to share such definitions for their methodologies enables others to apply the same processes to different datasets, facilitating result comparison and broader applicability.

Our approach facilitates reproducibility across diverse data sources, which is essential for federated analysis of fragmented datasets. Achieving interoperability among datasets is a crucial requirement for federated machine learning applications, and our solution offers a transparent and traceable pipeline to accomplish this goal (31). Additionally, it enables validation for robustness, bias, and fairness across different sites, thereby enhancing the reliability and integrity of AI models deployed in healthcare settings. The framework and its implementation serve as an implementation guideline for EHDS vision, tackling how data sets across different sites in Europe can be harmonized and aggregated for secondary use purposes while also ensuring traceability and end-to-end transparency fulfilling the requirements of AI-Act.

### 4.2 Limitations and future work

Currently, the definition of Target Population, Feature Group, and Feature Set necessitates technical proficiency in crafting FHIR query and FHIRPath expressions. To enhance user accessibility and usability, we intend to augment the onfhir-feast implementation with a graphical user interface. This interface will empower users to define FHIR query and FHIRPath expressions through visual expression builders, streamlining the process and reducing the reliance on technical skills.

The scope of the pipeline and implementation is limited to tabular datasets production. As foundational models are trained on raw data for generic purposes, researchers may prefer to provide directly the FHIR formatted data rather than a dataset tailored for a specific AI use case. However, still there is an important use case for generative AI where this pipeline can be useful. Recently, synthetic data generation for privacy preserving data sharing is one of the hot topics in healthcare AI. Our pipeline can be part of such setups where a common dataset definition can be used in different healthcare settings to extract harmonized datasets locally and then apply generative AI to create synthetic datasets that maintains the statistical properties of original datasets. Then these synthetic datasets can be shared, combined and used in model training, and development without exposing sensitive patient information.

Furthermore, we aim to expand the capabilities of onfhir-feast by providing visual tools to data scientists. These tools will facilitate querying and exploration of source data during target population selection and feature set preparation. By offering visualizations, data scientists can better assess the adequacy of the datasets provided by data sources in addressing the research question at hand, enhancing overall data exploration and analysis. The existing implementation already includes basic statistics, such as the number of missing values, maximum, minimum, and average for numeric values, as well as cardinalities, within the feature set documentation. Our objective is to expand the underlying language and enhance the onfhir-feast implementation to enable querying additional statistics about datasets.

We plan to leverage this extension for two primary purposes. Firstly, we aim to utilize it for constructing a metadata catalog, which will serve as a comprehensive repository of dataset statistics and characteristics. Secondly, we intend to employ it for developing data set exploration user interfaces tailored for data analysts. These interfaces will facilitate the assessment of data quality across various dimensions, including conformance, completeness, and plausibility, enabling users to evaluate the quality of datasets effectively.

## 5 Conclusion

In summary, the proposed methodology and models offer significant contributions to the ML research community in healthcare by establishing standardized, transparent, and technology-agnostic dataset definitions. These definitions not only characterize the datasets themselves but also delineate the procedures for compiling them from Electronic Health Record (EHR) systems via standard FHIR interfaces. This innovative approach represents a crucial step towards establishing best practices for data harmonization. By creating reusable, transparent, and shareable dataset definitions, it addresses a critical need in setting up federated data sharing environments for the secondary use of EHR data, such as the European Health Data Spaces initiative. By promoting interoperability and standardization, these methodologies pave the way for more efficient and effective ML research in healthcare, ultimately leading to improved patient outcomes and advancements in medical knowledge.

## Data availability statement

The datasets presented in this study can be found in online repositories. The names of the repository/repositories and accession number(s) can be found below: https://github.com/aiccelerate/common-data-model, https://github.com/aiccelerate/data-extraction-suite.

## Author contributions

TN: Writing−original draft, Software, Methodology, Formal analysis, Conceptualization. AS: Writing−original draft, Software, Methodology, Conceptualization. SG: Writing−review and editing, Software, Methodology, Conceptualization. CH: Writing−review and editing, Resources, Investigation. PG-C: Writing−review and editing, Resources, Investigation. AM: Writing−review and editing, Resources, Investigation. AE: Writing−review and editing, Resources, Investigation. GE: Writing−original draft, Methodology, Conceptualization.
