# Supplementary material for: A scalable and transparent data pipeline for AI-enabled health data ecosystems
Source: Front Med (Lausanne). 2024 Jul 30;11:1393123. doi: 10.3389/fmed.2024.1393123 (PMC11321077; doi:10.3389/fmed.2024.1393123)
Supplement: Supplementary file 1 [file Table_1.docx]

Supplementary Material

# Supplementary Figures and Tables

## Supplementary Tables

*Supplementary Table 1 List of features and outcome variables for the case study – predicting complications after cardiac surgeries*

| Features | Description |
| --- | --- |
| patient_demographics | |
| gender | Gender of the patient (male \| female \| other \| unknown) |
| age | Age of the patient at that time |
| episodes | |
| los | Length of stay (# of days) in hospital until that time |
| surgeries | |
| main_numberOfHours  AfterSurgery_X  X = [first, last] | Number of hours passed after first and latest surgery within the episode |
| main_mainProcedure  Code_X  X = [first, last, set] | ICD-10-PCS code for the main procedure performed in first and last surgery as well as set of codes for all surgeries performed in this episode until that time. |
| main_ccsCategory_X  X = [first, last, set] | Categorization of main procedure in terms of Clinical Classification Software (CCS)[32] performed in latest, first and all surgeries in this episode until that time. |
| main_classLevelY_X  X = [first, last, set]  Y = [1,2,3,4] | Class level 1,2,3,4 of ICD-10-PCS code for the main procedure performed in first, last and all surgeries in this episode until that time. |
| main_duration_X  X = [first, last, set] | Duration of first and latest surgery as well as sum of all surgeries in this episode until that time. |
| main_Y_X  X = [last, max]  Y=[aristotle, sts, rachs1] | Risk scores i.e. Aristotle score for congenial heart surgery[33], the Society of Thoracic Surgeons-European Association for Cardio-Thoracic Surgery risk score (STS)[34], the Risk Adjustment for Congenial Heart Surgery (RACHS1) for the latest performed surgery and the maximum score for within all surgeries. |
| main_isY_X  X = [last, any]  Y = [Extubated, Defibrilated] | Whether patient is extubated and/or defibrillated in the latest surgery as well as any surgery in this episode until that time. |
| main_min  Temperature_X  X=[last, min] | Minimum body temperature measured in the latest surgery as well as the minimum one within all surgeries in this episode until that time. |
| main_Y_X  X=[last, sum]  Y=[cecTime, clampTime, arrestTime] | Duration of specific procedures in minutes; extracorporeal circulation procedure, placement of aortic cross clamp, Hypothermic circulatory arrest for the latest surgery and sum of those times for all surgeries in this episode until that time. |
| other_numberOfHours  AfterOperation | If performed, number of hours passed after the latest diagnostic procedure like catheterization or electrophysiological studies |
| other_mainProcedureCode_X  X = [last, set] | ICD-10-PCS code for the latest diagnostic procedure as well as set of codes for all such procedures in this episode until that time. |
| surgeryEncounter | |
| numOfSurgeries  _count | Number of surgeries in this episode until that time |
| numOfNonSurgeryProcedures  _count | Number of diagnostic procedures related with cardiac surgery in this episode until that time |
| main_isElective_first | Whether first surgery in this episode is elective or not |
| otherProcedure | |
| code_X  X = [first, last, set] | Other surgical procedure codes (ICD-10-PCS) apart from main surgical procedure of latest, first and all surgeries in this episode until that time. |
| classLevelY_X  X = [first, last, set]  Y = [1,2,3,4] | 1st,2nd,3rd, and 4th level classification of ICD-10-PCS codes of other surgical procedure of latest, first and all surgeries in this episode until that time. |
| condition | |
| icd10Code_set | Set of ICD-10 diagnosis codes for patients until that time point. |
| icd10Code3Digits_set | Set of first 3 digits ICD-10 diagnosis codes for patients until that time point. |
| lab  Y = [hemoglobin, hematocrit, leucos,…] | |
| Y_value_pEET_X  X = [last, delta, rate] | For a specified list of lab tests (38 lab tests) that may be clinically important after cardiac surgeries, the latest value measured as well as the change in the value (difference with the previous one) and rate of change per day. |
| Y_interpretation_pEET | Code (HL7 ObservationInterpretationNormality codes) for indicating interpretation of latest lab result Y |
| Y_X_pEET  X = [isNormal, isHigh, isLow] | Whether the latest lab result Y is in normal range and high or low according to this range |
| icuOrWardStay | |
| numOfHoursInCurrent | Number of total hours in current location (ward or ICU) since admission to this location |
| ward_numOfHours_sum | Number of total hours passed in a ward after first surgery until that time point. |
| icu_encounterId_count | Number of ICU admissions in this episode until that time point. |
| icu_isCurrentlyInICU_any | Whether patient is in ICU at that time point. |
| icu_XSurgery_any  X = [before, after] | Number of ICU admissions before and after the first surgery in this episode |
| icu_numOfHoursXSurgery  _sum  X = [before, after] | Number of total hours in ICU before the first surgery and after the first surgery in this episode until that time point. |
| visscores | |
| withinX_max  X = [Or, 24h, 48h] | Post-operation, 24h and 48h Vasoactive Inotrope Score[35] values |
| vitalsign, bloodpressure  X=[ stddev, avg, max, min, median, kurtosis, skewness]  Y = [bodyTemprature, SPO2, heartrate, respiratoryRate, systolic, diastolic] | |
| bodyHeight_value | Latest body height measurement |
| bodyWeight_value | Latest body weight measurement |
| Y_value_pZ_X  Z = [2h, 4h, 8h] | Aggregations (i.e. standard deviation, average, maximum, minimum, median, kurtosis, skewness) of last (2,4 and 8)-hour time windows for each vital sign measurement |
| Y_value_Z_w1h_X  Z=[l1, l2, l3] | Aggregations of last 3 1-hour time windows for each vital sign measurement |
| medications | |
| Y_isGiven_p1d_any  Y=[metabolism, antithrombotic, …] | If a certain medication category, in terms of a specific categorization of ATC code based on clinical relevance, is given in last 24 hours.  ATC Code sections/groups used; A, B0, B02, B03, B05, C01, C02, C03, C05, C07, C08, C09, D, G, H01, H02, H03, H04, J01, J02, J04, J05, J06, J07, M01, M03, M04, N01, N02, N3, N05, N06, N07, P02, R03, R06, S, V |
| inotropics_any | If inotropics is given in the latest main surgery |
| complication | |
| other_exists_p8h_any | Whether any complication occurs in the last 8 hours |
| other_code_count | Number of complications after first surgery until that time |
| unexpectedICU_code_count | Number of unexpected ICU admissions due to complications until that time |
| Outcome Variables | |
| complication | |
| exists_f_any | Whether any complication occurs after that time point |
| minutesUntil_f | Number of minutes until first complication |
| unexpectedICU_exists_f_any | Whether any unexpected ICU admission occurs after that time point |
| unexpectedICU_minutesUntil_f | Number of minutes until first unexpected ICU admission |
| nX_w1h_any  X=[1-8] | Whether patient has a complication in the next Xth 1-hour period  e.g. 2ànext 2^nd^ hour period |
| unexpectedICU_nX_w1h_any  unexpectedICU_n1_w1h_any | Whether patient has a unexpected ICU admission in the next Xth 1-hour period |

References

32. UMLS Metathesaurus,. CCS (Clinical classifications software) - synopsis. (2024).

Available online at: https://www.nlm.nih.gov/research/umls/sourcereleasedocs/

current/CCS/index.html (accessed February 22, 2024).

33. Lacour-Gayet F, Clarke D, Jacobs J, Gaynor W, Hamilton L, Jacobs

M, et al. The Aristotle score for congenital heart surgery. Semin Thorac

Cardiovasc Surg Pediatr Card Surg Annu. (2004) 7:185–91. doi: 10.1053/J.PCSU.2004.

02.011

34. O’Brien S, Jacobs J, Pasquali S, Gaynor J, Karamlou T, Welke K, et al. The

society of thoracic surgeons congenital heart surgery database mortality risk model:

Part 1—statistical methodology. Ann Thorac Surg. (2015) 100:1054–62. doi: 10.1016/j.

athoracsur.2015.07.014

35. Belletti A, Lerose C, Zangrillo A, Landoni G. Vasoactive-inotropic score:

Evolution, clinical utility, and pitfalls. J Cardiothorac Vasc Anesth. (2021) 35:3067–77.

doi: 10.1053/J.JVCA.2020.09.117
